# Comparative transcriptomic analysis uncovers the complex genetic network for resistance to *Sclerotinia sclerotiorum* in *Brassica napus*

**DOI:** 10.1038/srep19007

**Published:** 2016-01-08

**Authors:** Jian Wu, Qing Zhao, Qingyong Yang, Han Liu, Qingyuan Li, Xinqi Yi, Yan Cheng, Liang Guo, Chuchuan Fan, Yongming Zhou

**Affiliations:** 1 National Key Laboratory of Crop Genetic Improvement, Huazhong Agricultural University, Wuhan 430070, China

## Abstract

Sclerotinia stem rot caused by *Sclerotinia sclerotiorum* is one of the most devastating diseases in many important crops including *Brassica napus* worldwide. Quantitative resistance is the only source for genetic improvement of *Sclerotinia*-resistance in *B. napus*, but the molecular basis for such a resistance is largely unknown. Here, we performed dynamic transcriptomic analyses to understand the differential defense response to *S. sclerotiorum* in a resistant line (R-line) and a susceptible line (S-line) of *B. napus* at 24, 48 and 96 h post-inoculation. Both the numbers of and fold changes in differentially expressed genes in the R-line were larger than those in the S-line. We identified 9001 relative differentially expressed genes in the R-line compared with the S-line. The differences between susceptibility and resistance were associated with the magnitude of expression changes in a set of genes involved in pathogen recognition, MAPK signaling cascade, WRKY transcription regulation, jasmonic acid/ethylene signaling pathways, and biosynthesis of defense-related protein and indolic glucosinolate. The results were supported by quantitation of defense-related enzyme activity and glucosinolate contents. Our results provide insights into the complex molecular mechanism of the defense response to *S. sclerotiorum* in *B. napus* and for development of effective strategies in *Sclerotinia*-resistance breeding.

The white mold fungus *Sclerotinia sclerotiorum* (Lib.) de Bary is a necrotrophic pathogen that infects more than 400 plant species, including important oil crops such as oilseed rape, soybean and sunflower^1^,^2^. Sclerotinia stem rot (SSR) of oilseed rape (*Brassica napus*) caused by *S. sclerotiorum* is the most devastating disease of this important oil crop in Australia, North America, Europe, India and China^3^. Yield losses in oilseed *Brassicas* (which includes oilseed rape and mustard (*B. juncea*)) vary between 5 and 100%^4^. Moreover, the oil content and quality of the seed are significantly reduced after infection^5^. The control of the *S. sclerotiorum* epidemic has been a great challenge in agronomic practice due to the wide host range and the pathogen’s survival capacity for long periods as sclerotia. Compared with cultural practices and fungicide application, breeding and cultivating resistant varieties are the most efficient, economic and environmentally friendly approaches to control SSR in oilseed rape and other crops^3^,^6^,^7^. Therefore, a better understanding of the genetic and molecular basis of the interactions of *S. sclerotiorum* with its host crops is essential for the effective breeding of *Sclerotinia*-resistant varieties.

To cope with pathogens, plants have evolved highly sophisticated immune systems that include pathogen-associated molecular pattern (PAMP)-triggered immunity (PTI) and effector-triggered immunity (ETI)^8^. A typical plant immune response may include a series of consecutive reactions from pathogen recognition, signal transduction, and hormone signaling pathways to downstream defense responses (e.g., the production of antimicrobial compounds)^9^. However, our understanding of the steps for the host plant immune response against *S. sclerotiorum* remains limited.

For pathogen recognition, PTI uses transmembrane pattern recognition receptors (PRRs) that respond to PAMP, while ETI uses resistance proteins that recognize pathogen effectors^8^. However, no PRRs or R proteins involved in recognizing *S. sclerotiorum* in host plants have been reported thus far. The immune responses in host plants are triggered through mitogen-activated protein kinase (MAPK) signaling cascades upon stimuli from pathogens^10^. As direct targets of MAP kinases, WRKY transcription factors (TFs) play broad and pivotal roles in regulating defenses^11^. Several studies have shown that MAPK signaling cascades and WRKY TFs are important in the defense responses against *S. sclerotiorum*. Overexpression of *BnMPK4*^12^ and *BnWRKY33*^13^ in *B. napus* and of *AtWRKY28* and *AtWRKY75* in *Arabidopsis*^14^ can enhance the resistance to *S. sclerotiorum*. Moreover, 5 *BnMAPKKKs*^15^, 3 *BnMKKs*, 3 *BnMPKs*^16^ and 13 *BnWRKYs*^17^ were induced significantly by *S. sclerotiorum*. Genome-wide analyses of MAPK signaling and WRKY genes that are responsive to *S. sclerotiorum* in *B. napus* or in other crops are lacking.

Downstream of PTI or ETI activation, three plant hormones, i.e., salicylic acid (SA), jasmonic acid (JA) and ethylene (ET), are recognized as key players in the regulation of plant defense responses^18^,^19^. Microarray expression profiling showed that genes associated with JA and ET signaling pathways are responsive to *S. sclerotiorum* infection in *B. napus*^20^,^21^,^22^. These results are consistent with the general model that SA is involved in the activation of defense responses against biotrophic and hemi-biotrophic pathogens, whereas JA and ET are associated with defense responses against necrotrophic pathogens^18^. However, two recent studies showed that SA might play a positive role in the defense responses of *B. napus* against *S. sclerotiorum*^23^,^24^. Conflicting results regarding whether SA is involved in the defense responses of *Arabidopsis* to *S. sclerotiorum* were also obtained^25^,^26^,^27^. Further studies are required to better understand the plant hormonal regulation of the defense responses against *S. sclerotiorum*.

After a series of signal transductions, active plant defenses are induced to restrict pathogen development, which can be accomplished through the production of antimicrobial compounds and reinforcement of cell walls. Constitutive overexpression of a endochitinase gene in *B. napus*^28^ and a *B. napus* polygalacturonase inhibitor protein (PGIP) gene (*BnPGIP2*) in *Arabidopsis*^29^ can enhance the resistance to *S. sclerotiorum*. In *Arabidopsis*, genes associated with the formation of the secondary metabolites camalexin and glucosinolate were induced in leaves challenged with *S. sclerotiorum*, and mutant lines deficient in camalexin, indole, or aliphatic glucosinolate biosynthesis were hypersusceptible to *S. sclerotiorum*^30^. Moreover, the biosynthesis of monolignol could reinforce plant cell walls and is associated with the resistance to *S. sclerotiorum* in *Camelina sativa*^31^.

Earlier studies characterized the transcriptomic changes in *B. napus* during the defense responses to *S. sclerotiorum* using *Arabidopsis*- or *B. napus*-specific oligonucleotide microarrays^20^,^21^,^22^. However, the limited information collected through microarrays cannot provide a comprehensive understanding of the defense responses because of the complicated *B. napus* genome^32^,^33^. Next-generation sequencing technologies enable researchers to study whole transcriptomes and offer greater power to distinguish homologous genes and to detect low- and high-abundance transcripts^34^. The recent release of the *B. napus*^32^ genome sequences, together with next-generation sequencing technologies, provides an unprecedented opportunity to monitor the transcriptomic profiles of defense responses to *S. sclerotiorum* in *B. napus*.

In this study, we performed a transcriptomic analysis of resistant and susceptible *B. napus* lines to investigate the defense responses to *S. sclerotiorum* using in-depth RNA sequencing (RNA-seq). We identified and characterized the relative differentially expressed genes between resistant and susceptible lines. Important genes or processes that may be involved in the pathogenicity of *S. sclerotiorum* were identified through monitoring the transcriptomic changes in *S. sclerotiorum*. Meanwhile, we verified the transcriptomic results by monitoring defense-related enzyme activity and metabolite content. Our data provide insights into the genetic and molecular basis of the resistance to *S. sclerotiorum* in *B. napus*.

## Results

### Resistance to *S. sclerotiorum* in rapeseed is characteristic by slower lesion expansion after infection

**Figure 1:**
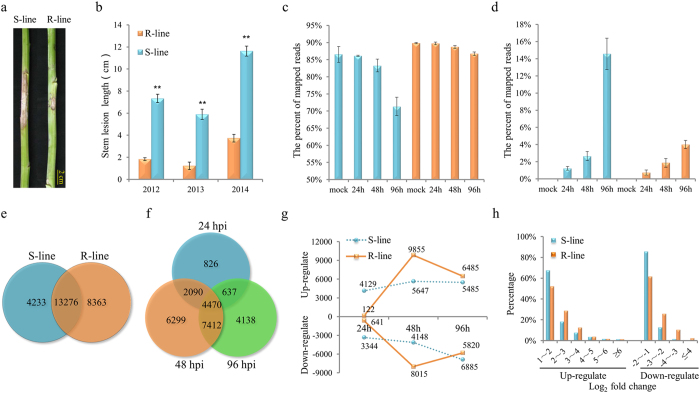
Phenotypic characterization and DEGs identification in the R- and S-lines after *S. sclerotiorum* infection. (**a**) Disease lesions on the stems of the two lines at 7 d post-inoculation in 2013. (**b**) Lesion length of the two lines at 7 d post-inoculation in 2012–2014. The bars represent the standard error (n = 10). ** indicates a significant difference at P < 0.01 (two tailed T-test). (**c**,**d**) Proportion of the clean reads of each sample mapped to the *B. napus* (**c**) or *S. sclerotiorum* (**d**) genome sequence, the means of each sampling time point and of each treatment were calculated from three biological replicates. (**e**) In total, 21,639 and 17,509 DEGs were identified from the R- and S-lines, respectively, and 13,276 DEGs were identified in both lines. (**f**) Venn diagram showing the DEGs expressed at each of the three sampling time points (24, 48 and 96 hpi) of the two lines. The overlapping regions correspond to the number of DEGs present at more than one sampling point. (**g**) The number of DEGs that were up- or down-regulated at different time points in the two lines. (**h**) The fold change in the DEGs detected in the R- and S-lines at 48 hpi.

Two *B. napus* pure lines, J964 (resistant line, designated the R-line) and J902 (susceptible line, designated the S-line) were inoculated with the *S. sclerotiorum* isolate on the primary stem using mycelial agar plugs at the stage of flowering termination. Visible lesions appeared in the S-line at 72 h post-inoculation (hpi) and in the R-line at approximately 96 hpi. The lesions extended more rapidly in the S-line than in the R-line. After 7 days, the lesions on the R-line stems were significantly smaller than those on the S-line (Fig. 1a). The resistance assays were performed in three consecutive years (2012–2014) (Fig. 1b), and similar results were observed in different years, suggesting that the resistance difference between these two genotypes is highly stable.

### High quality RNA-seq data uncover the dynamic changes of gene expression in both the host plant and pathogen

RNA was isolated from the stems of the R- and S-lines at 24, 48 and 96 h after *S. sclerotiorum* or mock inoculation. Three biological replicates were sampled. In total, 24 RNA samples were subjected to paired-end RNA sequencing, and 885.5 million clean reads were obtained with an average of 36.9 million reads (3.3 Gb) for each sample (Supplementary Table 1). The clean reads of each sample were mapped to the *B. napus*^32^ and *S. sclerotiorum*^35^ genome sequences. For the mock-inoculated samples from the R- and S-lines, 89.8% and 86.5% (average of three biological replicates) of the reads, respectively, were mapped to the *B. napus* genome sequences (Fig. 1c). No reads were mapped to the *S. sclerotiorum* genome sequence, as expected (Fig. 1d). Furthermore, on average, 85.3% of the reads from all samples could be mapped to the *B. napus* genome sequence. Of these mapped reads, approximately 90% matched uniquely (Supplementary Table 1).

After inoculation with *S. sclerotiorum*, the proportion of the reads mapped to the *S. sclerotiorum* genome sequence increased over time for both the R- and S-lines. Meanwhile, the proportion of the reads mapping to the *B. napus* genome sequence decreased (Fig. 1c,d). In total, 28.2 million clean reads from all inoculated samples were mapped to the *S. sclerotiorum* genome (Supplementary Table 1). The proportions of the reads mapping to the *S. sclerotiorum* genome sequence in the S-line were significantly higher than those in the R-line at all sampling points. The rate of increase in the proportion of reads mapping to the *S. sclerotiorum* genome was higher in the S-line (Fig. 1d). Thus, the data suggest that *S. sclerotiorum* could infect and propagate in the S-line more easily than in the R-line. This difference observed through RNA-seq was consistent with the phenotypic difference between these two lines.

Using a FPKM (fragments per kilobase of transcript per million fragments mapped) cutoff value of 1 from the average of three biological replicates, 42,813 and 41,385 genes were detected to express in the mock-inoculated samples of the R- and S-lines, respectively, which accounted for 41–42% of the 101,040 annotated *B. napus* genes (Supplementary Table 2). In addition, there were 13,556 and 15,180 in the mock-inoculated samples of R- and S-lines, respectively, with FPKM values between 0.1 and 1 (Supplementary Table 2), which were detected as weakly expressed genes partly due to many genes being tissue-specific and/or development-specific. Pearson correlation coefficients (R) between each pair of biological replicates at different sampling time points and under different treatments for both the R- and S-lines were high (R > 0.95 in most cases; Supplementary Fig. 1), indicating that the RNA-seq data among biological replicates were of high quality.

### Transcriptomic changes in response to *S. sclerotiorum* inoculation

To investigate the differential responses to *S. sclerotiorum* inoculation between the R- and S-lines, we identified the differentially expressed genes (DEGs) between the inoculated and mock-inoculated samples. In total, 21,639 and 17,509 DEGs were identified in the R- and S-lines, respectively, at all sampling time points (Fig. 1e and Supplementary Table 3). Among these, 13,276 DEGs were present in both lines, while 8,363 and 4,233 were R-line specific and S-line specific, respectively (Fig. 1e). These results indicate that *S. sclerotiorum* infection can cause a dramatic change in host’s plant transcription (∼20% of total annotated genes in *B. napus* genome), and the defense responses from resistant and susceptible lines might be significantly different.

Considering these two lines together, we detected 8,023, 20,271 and 16,657 DEGs at 24, 48 and 96 hpi, respectively (Fig. 1f). Among all DEGs, 14,609 DEGs (56.5%) were present at two or three time points. The remaining DEGs were sampling-point specific, with 826, 6299 and 4,138 DEGs specific for 24, 48 and 96 hpi, respectively (Fig. 1f). The transcriptomic changes occurred most dramatically at 48 hpi.

Subsequently, we examined the DEGs that were up- or down-regulated at different sampling time points in these two lines. At 24 hpi, 4,129 genes were up-regulated and 3,344 genes were down-regulated in the S-line, while only 122 genes were up-regulated and 641 genes were down-regulated in the R-line (Fig. 1g), suggesting that an easier establishment of *S. sclerotiorum* infection (Fig. 1a,b) may lead to earlier transcriptomic response in the S-line compared with the R-line. At 48 hpi, the numbers of up-regulated genes and down-regulated genes detected in the R-line were 9855 and 8015, respectively, almost twice the number of the up- and down-regulated genes in the S-line (Fig. 1g). Furthermore, there were more genes with ≥ 4-fold change (up- or down-regulated) in the R-line than in the S-line (Fig. 1h). Hence, both the DEG numbers and fold changes in the R-line exhibit a more intense defensive response than in the S-line at 48 hpi. At 96 hpi, more up-regulated genes (6,485 vs 5,485) and fewer down-regulated genes (5,820 vs 6,885) were present in the R-line compared with those in the S-line (Fig. 1g).

**Figure 2:**
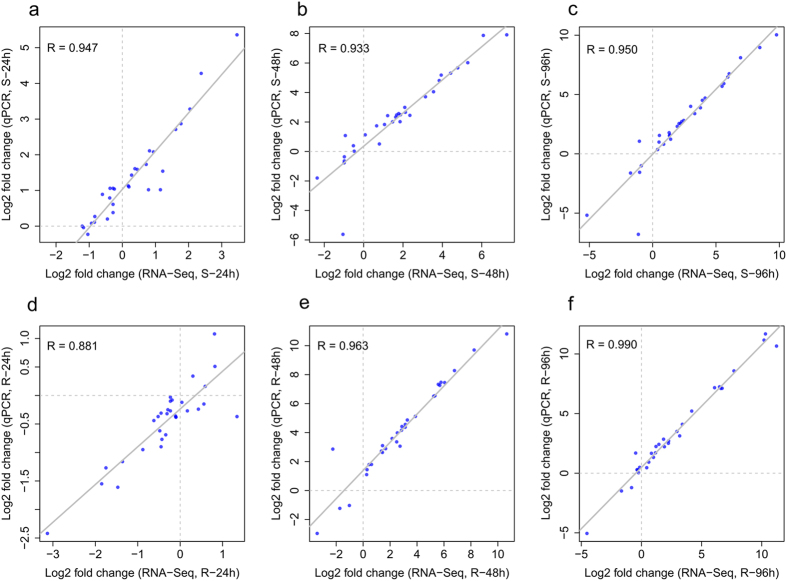
Correlation of gene expression ratios obtained by qPCR and RNA-seq. The qPCR log_2_ value of the expression ratio (inoculated/mock-inoculated) (y-axis) was plotted against the value from the RNA-seq (x-axis). All qPCR data were collected from three biological replicates and four technical replicates for each sample. S-line: (a) 24 hpi; (**b**) 48 hpi; (**c**) 96 hpi. R-line: (**d**) 24 hpi; (**e**) 48 hpi; (f) 96 hpi.

To validate the data obtained by RNA-seq, 31 genes were selected for qPCR assays (Supplementary Table 4). The relative expression levels measured by qPCR were converted to fold changes (inoculated/mock-inoculated) to enable a direct comparison with RNA-seq data. The results obtained using the two techniques highly correlated for both lines at the three sampling time points (R = 0.881–0.990, Fig. 2), demonstrating the reliability of the data produced through RNA-seq.

### Identification of relative differentially expressed genes in the R-line

To identify the important genes responsible for *Sclerotinia* resistance in the R-line, we divided all 25,872 DEGs in these two lines into 36 clusters based on their expression patterns using Genesis based on the K-means clustering method^36^ (Supplementary Fig. 2). The DEGs in most clusters showed a similar pattern between the R- and S-lines, except for cluster 12, which was consistently up-regulated in the R-line and down-regulated in the S-line. Interestingly, the fold changes in many DEGs were more dramatic in the R-line than in the S-line at some time points, including up-regulated genes in clusters 1, 6, 7, 10, 14, 16, 19, 21, 23, 25 and 32 and down-regulated genes in clusters 4, 5, 9 and 18 (Supplementary Fig. 2).

To better understand these genes with different expression patterns or fold changes between the R- and S-lines, we introduced the concept of relative differentially expressed genes (RDEGs). A gene was defined as a RDEG in the R-line (compared with the S-line) when it was detected as a DEG in the R-line and when its fold change was more than 2-fold than that in the S-line at the corresponding time points.

**Figure 3:**
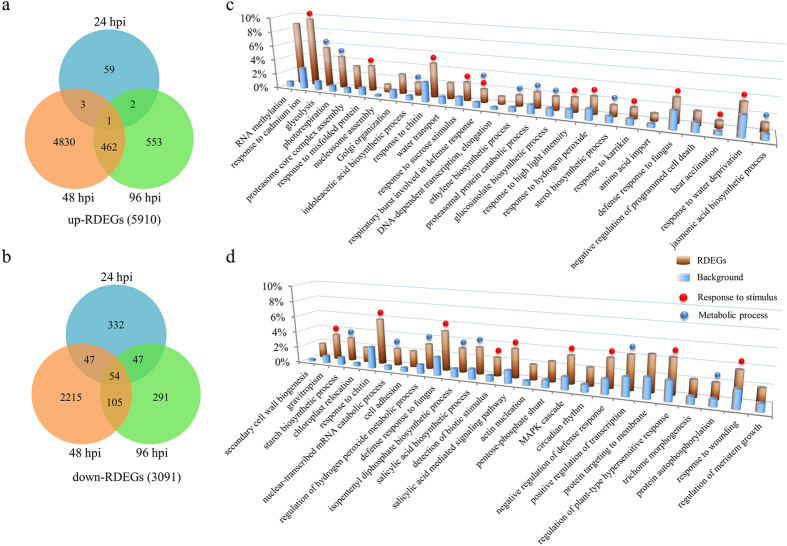
Identification and GO enrichment analysis of RDEGs that may be responsible for the *Sclerotinia* resistance of the R-line. (**a**,**b**) Venn diagram showing the up-RDEGs (**a**) and down-RDEGs (**b**) in each of the three sampling time points (24, 48 and 96 hpi). (**c**,**d**) Biological process categorization of the RDEGs based on GO enrichment analysis. The y-axis is the percentage of genes mapped by the term, representing the abundance of the GO term. The percentage for the input list is calculated by the number of genes mapped to the GO term divided by the number of all genes in the input list. The same calculation was applied to the reference list (background) to generate its percentage. Terms containing over 50 up-RDEGs (**c**) or 50 down-RDEGs (**d**) are shown.

In total, 5,910 up-regulated RDEGs (up-RDEGs) and 3,091 down-regulated RDEGs (down-RDEGs) in the R-line were identified (Fig. 3a,b and Supplementary Table 5). Most up- or down-RDEGs were detected at 48 hpi (5,296 and 2,421, respectively, Fig. 3a,b), suggesting that the R-line defense responses were more strongly activated at 48 hpi.

### Functional classifications of RDEGs

For gene function annotation, all 101,040 *B. napus* genes were searched against the Nr and InterPro databases, and 90,699 (89.8%) and 85,570 (84.7%) genes were annotated in these two databases, respectively. Gene ontology (GO) terms were annotated by merging Blast2GO and InterPro annotation results, and 81,759 (80.9%) genes were assigned to at least one GO term.

To gain insights into the functionality of the RDEGs in the R-line in response to *S. sclerotiorum* infection, we performed GO enrichment analysis using Blast2GO^37^. With a cutoff value of FDR < 0.01, 231 and 137 enriched GO classes were identified for up-RDEGs and down-RDEGs, respectively. These GO classes were included in following three categories: biological process, cellular component and molecular function (Supplementary Table 6). We focused on the significantly enriched biological process terms that contained over 50 up-RDEGs or down-RDEGs (Fig. 3c,d).

Most of the enriched GO terms belonged to two secondary categories of biological processes, i.e., response to stimulus and metabolic process (Fig. 3c,d), suggesting that the expression differences in the genes involved in these two biological processes may play important roles in the differential responses between the R- and S-lines after inoculation. The genes related to various defense responses were significantly enriched in up-RDEGs, such as those genes responding to chitin, fungus, cadmium ion and hydrogen peroxide (Fig. 3c). Interestingly, the glycolysis process was enriched in up-RDEGs (Fig. 3c), while the starch biosynthetic process was enriched in down-RDEGs (Fig. 3d), indicating that the R-line may be more capable to coordinate the two metabolic process to increase energy supply for defense responses than the S-line. Both ET and JA biosynthetic processes were enriched in up-RDEGs (Fig. 3c), while the SA biosynthetic process was enriched in down-RDEGs (Fig. 3d), indicating that the resistance to *S. sclerotiorum* might be positively regulated by JA and ET and negatively regulated by SA. In addition, the glucosinolate biosynthetic process was also enriched in up-RDEGs (Fig. 3c). The GO term analyses thus provide promising candidate genes underpinning R-line resistance; these genes are examined in detail in the following sections.

### Identification of important genes involved in the defense network responsive to *S. sclerotiorum* infection

To survive the invasion of pathogens, plants require an effective response to restrict the further propagation of the pathogen. Such a quick reaction heavily rely on the gene network involving recognition, MAPK signaling cascades, WRKY transcription regulation, hormone signaling pathways, defense-related protein production and secondary metabolite biosynthesis.

### Recognition of S. sclerotiorum by RLKs or by R proteins

Membrane-localized receptor-like kinases (RLKs) are well-characterized plant PRRs involved in pathogen recognition^38^, but their roles in SSRs remain unclear. The *Arabidopsis* genome contains over 300 RLKs (http://www.arabidopsis.org/browse/genefamily/Receptor_kinase.jsp). We identified over 1,200 homologs of these genes in the *B. napus* genome (Supplementary Table 7). Of 57 RLK genes induced in the R- and S-lines (Supplementary Table 8), 19 were up-RDEGs in the R-line. Seven of these 19 up-RDEGs were induced in the R-line but not in the S-line (Table 1). Most of the 19 up-RDEGs encode leucine-rich repeat RLK (LRR-RLK)-, wall-associated kinase-like (WAKL)-, L-type lectin receptor- and CRINKLY4-related kinases.

Whether any R-genes are associated with the resistance to *S. sclerotiorum* in *B. napus* is unknown. In total, 425 nucleotide binding site leucine-rich repeat (NBS-LRR) genes have been identified in the *B. napus* genome^32^. Among the 6 NBS-LRR genes induced in the R- and S-lines (Supplementary Table 8), 3 were up-RDEGs in the R-line (*BnaC06g24010D* and *BnaA09g21180D* were specifically induced in the R-line, and *BnaA03g38380D* was induced more in the R-line) (Table 1). Notably, most of the differentially expressed RLK or NBS-LRR genes were induced at 24 hpi in the S-line but at 48 hpi in the R-line (Table 1 and Supplementary Table 8). This observation may be attributed to the earlier establishment of infection in the S-line.

### Signal transduction by the MAPK cascade

**Figure 4:**
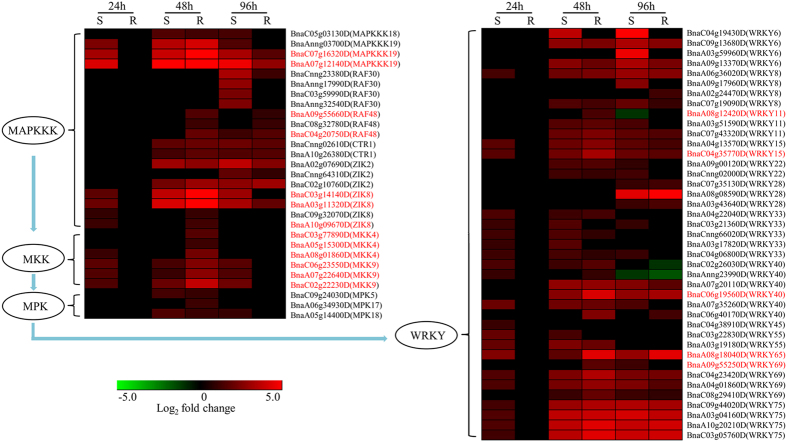
Heat maps of the MAPKKK, MKK and MPK genes and WRKY transcription factors induced after *S.* sclerotiorum infection. Genes in red are up-RDEGs.

Extracellular stimuli from pathogens sensed by receptors activate distinct MAPK cascades, which minimally consist of a MAPKKK-MKK-MPK module^10^. In total, 258 MAPKKK, 29 MKK and 78 MPK genes were identified in the *B. napus* genome based on 80 MAPKKK, 10 MKK and 20 MPK genes in *Arabidopsis*^39^ using the BlastP program (Supplementary Table 7). To identify important MAPK signaling cascades responsive to *S. sclerotiorum*, we focused on the expression changes in all MAPKKK, MKK and MPK genes in *B. napus*. In total, 20 MAPKKK, 6 MKK and 3 MPK genes were induced in the R- and S-lines (Fig. 4). Among these induced genes, 7 MAPKKK genes (2 *MAPKKK19*, 2 *RAF48* and 3 *ZIK8*) and 6 MKK genes (3 *MKK4* and 3 *MKK9*) were up-RDEGs (Fig. 4). In the R-line, the most strongly induced MAPKKK genes were two copies of *MAPKKK19* (*BnaA07g12140D* and *BnaC07g16320D*), which were up-regulated by 161- and 44-fold, respectively, at 48 hpi. Moreover, the most strongly induced MKK genes were three copies of *MKK9* (*BnaC06g23550D*, *BnaA07g22640D* and *BnaC02g22230D*) that were up-regulated by 6-, 7- and 14-fold at 48 hpi, respectively (Fig. 4 and Supplementary Table 3).

### WRKY transcription factors responsive to S. sclerotiorum

*Arabidopsis* has 72 expressed WRKY genes (http://www.arabidopsis.org/browse/genefamily/WRKY.jsp). We identified 289 homologs of these genes in the *B. napus* genome (Supplementary Table 7). In total, 41 WRKY genes were induced in the R- and S-lines, including several copies of *WRKY6*, *8*, *11*, *15*, *28*, *33*, *40*, *69* and *75*. Four copies of *WRKY75* were the most strongly induced WRKY genes in both lines (Fig. 4). Six up-RDEGs were detected, including *BnaA08g12420D* (*WRKY11*), *BnaC04g35770D* (*WRKY15*), *BnaC06g19560D* (*WRKY40*), *BnaC06g40170D* (*WRKY40*), *BnaA08g18040D* (*WRKY65*) and *BnaA09g55250D* (*WRKY69*) (Fig. 4). We also identified 70 down-regulated WRKY genes in these two lines, including all six copies of *WRKY70* (Supplementary Fig. 3), an activator of SA-induced genes and a repressor of JA-responsive genes that integrates signals from these mutually antagonistic pathways^40^. Interestingly, five copies of *WRKY70* were down-RDEGs, suggesting that the SA signaling pathway might be more significantly down-regulated in the R-line compared to the S-line.

### Plant hormones in defense responses to S. sclerotiorum

**Figure 5:**
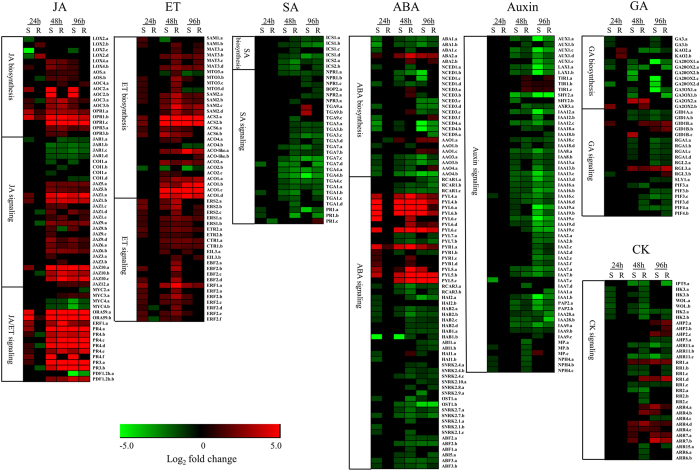
Plant hormones in defense responses to *S. sclerotiorum*. The heat map showed the up- or down-regulated of the major genes involved in the biosynthesis and signaling pathways of SA, JA, ET, ABA, auxin, GA and CK in the R- and S-lines. All hormone biosynthesis and signaling pathway genes identified in *B. napus* are listed in detail in Supplementary Table 9.

The biosynthesis of a number of hormones, including SA, JA, ET, abscisic acid (ABA), and gibberellic acid (GA), was affected by *S. sclerotiorum* infection (Fig. 5 and Supplementary Table 9). For example, most of the genes involved in the biosynthesis of JA and ET were significantly up-regulated, while most of the SA, ABA and GA biosynthetic genes were significantly down-regulated (Fig. 5). By examining the major genes involved in the hormone signaling pathways, we found that the JA and ET signaling pathways were significantly induced, while the SA, ABA, GA, auxin and CK signaling pathways were noticeably inhibited (Fig. 5). Two major branches of the JA signaling pathway in *Arabidopsis* are the MYC branch, which is regulated by MYC-type TFs, and the ERF branch, which is regulated by ERF1 and ORA59^19^. We found that *ERF1*, *ORA59* and their regulated genes (such as *PDF1.2*, *CHIB* (*pathogenesis-related protein* (*PR*) *3*) and *HEL* (*PR4*)) were significantly up-regulated, while most of the *MYC* TFs were down-regulated, indicating that the ERF branch of the JA signaling pathway is associated with the defense responses to *S. sclerotiorum*. Interestingly, many important genes involved in the ERF branch of the JA signaling pathway were up-RDEGs, including one copy of *ERF1*, two copies of *CHIB* and three copies of *HEL* (Fig. 5 and Supplementary Table 9), highlighting the importance of the ERF branch in *Sclerotinia* resistance. Moreover, two copies of *LOX2* and one copy of *AOC4*, which are important for JA biosynthesis, were up-RDEGs (Fig. 5 and Supplementary Table 9). Important genes involved in ET biosynthesis (e.g., some copies of *SAM1*, *MTO3*, *SAM2*, *ACS2* and *ACO1*) and signaling pathway (e.g., some copies of *ERS2*, *EIL3*, *EBF2*, *ERF1* and *ERF2*) were up-RDEGs in the R-line, while important genes involved in SA biosynthesis (e.g., some copies of *ICS1* and *ICS2*) and in the SA signaling pathway (e.g., *TGA3*, *TGA1* and *PR1*) were down-RDEGs in the R-line (Fig. 5 and Supplementary Table 9). These results are consistent with the results of the GO enrichment analysis and thus suggest that JA and ET biosynthesis and signaling pathways were induced more significantly, while SA biosynthesis and signaling pathway were inhibited more strongly in the R-line.

### Genes encoding the defense-related proteins

Glucanase and chitinase are involved in the degradation of glucan and chitin, the primary structural components of fungal cell walls. Genes encoding two types of glucanases (β-1,3-endoglucanase and β-1,3-glucanase 2 (PR2)) were induced in the R-line, while only β-1,3-endoglucanase genes were induced in the S-line (Table 2). Among these induced glucanase genes, two β-1,3-endoglucanase genes (*BnaA01g17540D* and *BnaC01g21880D*) were most strongly induced and were up-RDEGs. At 48 hpi, these two genes were up-regulated by 831.7- and 190.0-fold in the R-line and by 64.9- and 39.9-fold in the S-line. The fold changes were even greater at 96 hpi; these genes were up-regulated by 2288.2- and 843.4-fold in the R-line and by 498.0- and 238.9-fold in the S-line (Table 2). PR3 and chitinase family genes encoding chitinase were strongly regulated. Remarkably, a greater number of chitinase family genes was induced in the R-line (8 copies) than in the S-line (5 copies) (Table 2). The degrees of up-regulation of all the chitinase family genes were much larger in the R-line than in the S-line at 48 and 96 hpi (five copies were up-RDEGs) (Table 2). The most strongly induced chitinase family gene was *BnaC04g09720D*, which was up-regulated by 1389.2-fold in the R-line and by 541.2-fold in the S-line (Table 2). Two copies of PR3 genes (*BnaA05g26640D* and *BnaC05g40680D*) were up-RDEGs. *BnaA05g26640D* was up-regulated by 344.9-fold in the R-line, but by only 54.2-fold in the S-line at 48 hpi (Table 2). Furthermore, two types of genes responding to chitin were also strongly induced, including six copies of PR4 (similar to the antifungal chitin-binding hevein protein genes) and four copies of legume lectin genes, most of which were up-RDEGs (3 PR4 and 4 legume lectin genes). For example, *BnaCnng78710D* was up-regulated by 5595.3-fold in the R-line but by only 831.7-fold in the S-line at 48 hpi (Table 2).

PGIPs are located in the plant cell wall and counteract the action of PGs to prevent cell wall degradation^41^. Seven copies of PGIP genes were induced after *S. sclerotiorum* inoculation. All seven PGIP genes were more strongly induced or only induced in the R-line at 48 hpi (six copies of PGIP genes were up-RDEGs) (Table 2). Furthermore, genes encoding PDF1.2b, PR5-like and osmotin proteins possessing antimicrobial activities were significantly more strongly induced in the R-line than in the S-line (Table 2).

**Figure 6:**
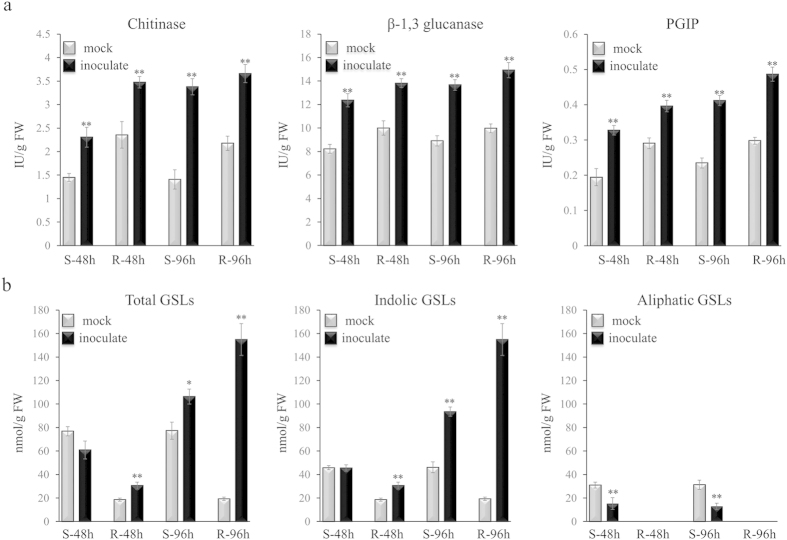
Quantifications of chitinase, β-1,3 glucanase, PGIP activity (**a**) and the glucosinolates content (**b**) in the R- and S-lines after *S. sclerotiorum* inoculation. The bars represent the standard error (n = 5). * and ** indicate a significant difference between inoculate and mock at P < 0.05 and P < 0.01 levels, respectively (two tailed T-test).

To verify the results of above transcriptomic analysis, we measured the activity of the three defense-related enzymes (chitinase, β-1,3 glucanase and PGIP). All the three enzymes appeared to be stimulated at both R- and S-lines after *S. sclerotiorum* inoculation (Fig. 6a). In R-line, the three enzymes activities were raised to high levels at 48 hpi, and then increased slightly from 48 hpi to 96 hpi, whereas their activities in S-line were relatively low compared with R-line both at 48 and 96 hpi (Fig. 6a). Therefore, the activities of the three defense-related enzymes were highly consistent with the difference in gene expression changes between the two lines (Table 2). In addition, the activities of the three enzymes were also higher in R-line than in S-line before inoculation (Fig. 6a), which may partially explained the difference between the R- and S-lines at the initial infection.

*Glucosinolate content was significantly enhanced in responsive to S. sclerotiorum infection.* 

**Figure 7:**
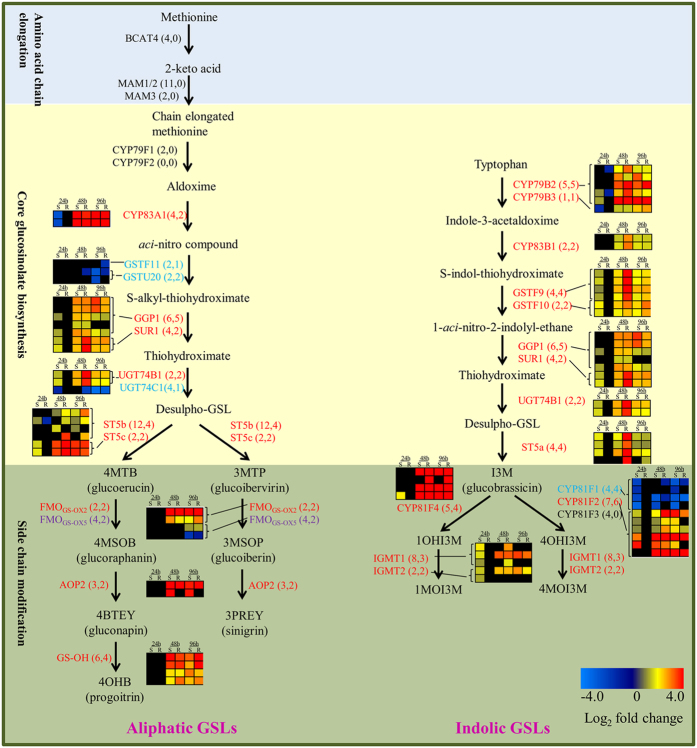
Whole-genome-wide comparison of genes involved in GSL biosynthesis pathways (adapted from Liu *et al.*^56^) in *B*. n*apus* and expression profiles of these genes in R- and S-lines defense responses to *S. sclerotiorum*. The copy number of GSL biosynthetic genes and the differentially expressed genes are listed in square brackets, respectively. Genes in red or blue indicate up-regulated or down-regulated genes, respectively. Genes in purple are those with both up-regulated and down-regulated copies, whereas genes shown in black are not differentially expressed. 1MOI3M: 1-methoxyindol-3-ylmethyl GSL; 1OHI3M: 1-hydroxyindol-3-ylmethyl GSL; 3MSOP: 3-methylsulfinylpropyl GSL; 3MTP: 3-methylthiopropyl GSL; 3PREY: 2-Propenyl GSL; 4BTEY: 3-butenyl GSL; 4MOI3M: 4-methoxyindol-3-ylmethyl GSL; 4OHB: 4-hydroxybutyl GSL; 4OHI3M: 4-hydroxyindol-3-ylmethyl GSL; 4MSOB: 4-methylsulfinylbutyl GSL; 4MTB: 4-methylthiobutyl GSL.

In this study, 214 GSL biosynthesis homologous genes were identified in *B. napus* using the 57 *Arabidopsis* GSL biosynthesis genes (Supplementary Table 10). To distinguish the roles of aliphatic GSL and indolic GSL, we analyzed the expression changes in the major genes involved in the biosynthesis of the two types of glucosinolates separately. Most of the indolic GSL biosynthesis genes were induced in both lines after *S. sclerotiorum* inoculation. Almost all copies of the genes involved in the core indolic GSL biosynthesis pathway were induced (Fig. 7). In contrast, the regulation of aliphatic GSL biosynthesis genes was more complicated. For example, the *BCAT4*, *MAM1/2* and *MAM3* genes, which are involved in amino acid chain elongation, had no significant expression changes, while the core glucosinolate biosynthesis genes *GSTF11*, *GSTU20* and *UGT74C1* were down-regulated, and *CYP79F1* genes had no significant expression changes. Furthermore, other up-regulated core glucosinolate biosynthesis genes were also involved in the regulation of indolic GSL biosynthesis (Fig. 7). More importantly, almost all copies of *CYP79B2*, *CYP83B1*, *GSTF9*, *GSTF10*, *SUR1*, *UGT74B1* and *ST5a*, which are involved in core indolic GSL biosynthesis, were up-RDEGs (Fig. 7), suggesting that indolic GSL biosynthesis was more intensely induced in the R-line than in the S-line, particularly at 48 hpi.

To verify above results, we measured the content of GSLs in the inoculated or mock-inoculated plants of the R- and S-lines, which are double-low (low erucic acid and low glucosinolates) lines having very low amount of aliphatic GSL. There were two aliphatic (2-hydroxy-3-Butenyl and 2-hydroxy-4-Pentenyl) and three indolic (4-hydroxy-indol-3-ylmethyl, Indol-3-ylmethyl and 4-methoxy-indol-3-ylmethyl) GSLs detectable (Supplementary Table 11). Because similar variation trends were observed between the two aliphatic GSLs or between the three indolic GSLs after inoculation, we only showed the content change of total aliphatic and total indolic GSLs. The aliphatic GSLs were only detected in the S-line, and the total content was significantly reduced after inoculation (Fig. 6b). All of the three indolic GSLs were detected in both lines. In the R-line, the total indolic GSLs content significantly increased at 48 hpi, and the increase was even more dramatic at 96 hpi. While in the S-line, the total indolic GSLs content showed no change at 48 hpi and a less dramatic increase at 96 hpi compare with the R-line (Fig. 6b). These results are consistent with the expression data and thus highlight the importance of indolic GSL biosynthesis in *Sclerotinia* resistance.

## Discussion

As a necrotrophic fungus with an extremely broad host spectrum, the pathogen *S. sclerotiorum* extracts nutrients from the dead cells killed before or during colonization. This disease imposes huge yield loss in oilseed rape and in other important crops worldwide each year. Unfortunately, no immune or highly resistant germplasm in *B. napus* and its close relatives has been identified thus far. Exploring the genetic resources and understanding the genotypic differences in resistance are urgently needed for developing an effective strategy for *Sclerotinia*-resistance breeding. In this study, we presented an in-depth transcriptomic analysis of the defense responses to *S. sclerotiorum* in resistant and susceptible *B. napus* lines. Our results revealed a complex and coordinated gene network conferring resistance differences between genotypes, further expanding our knowledge regarding how *B. napus* survives deadly *S. sclerotiorum* attacks.

Previously, a 70-mer oligo-microarray containing 26,000 annotated *Arabidopsis* genes was used to characterize the transcriptomic changes in response to *S. sclerotiorum* in *B. napus*^20^,^21^. However, the whole-genome duplication in *B. napus* makes it difficult to distinguish the expression patterns of homologous genes in *B. napus* by an *Arabidopsis*-specific microarray. Zhao *et al.*^22^ used a *B. napus*-specific 50-mer oligo-microarray to examine the transcriptional changes in response to *S. sclerotiorum* infection of *B. napus*. But the *B. napus*-specific microarray only represents 15,000 unique genes (14.8% of all *B. napus*genes). In our study, approximately 90% of mapped reads could be unambiguously mapped to unique regions of the *B. napus* genome (Supplementary Table 1). Only the uniquely mapped reads were considered for gene expression analysis, ensuring that the transcript abundances of homologous genes were measured accurately. By examining the expression of homologous genes, we were able to identify differential expression patterns between homologous genes. For example, we found that one copy of *PDF1.2b* (*BnaA07g32130D*) was up-regulated at 48 and 96 hpi, while another copy of *PDF1.2b* (*BnaA07g32150D*) down-regulated at 96 hpi, and other copies were not differentially expressed (Fig. 5 and Supplementary Table 5). Our study thus not only provided much larger volume of information but also revealed the novel patterns at homologous gene levels for the transcriptomic changes after *S. sclerotiorum* infection in allotetraploid *B. napus*.

To explore the mechanisms by which the R-line has superior *Sclerotinia* resistance over the S-line, we introduced the RDEGs in the R-line compared to the S-line based on the identification of the DEGs in the R- and S-lines. This comparison allowed us to minimize false-positive or false-negative results. For example, an R-line-specific DEG may not have a substantial difference between these two lines (e.g., choosing a gene with a 2-fold R-line change and a 1.8-fold S-line change would cause a false-positive result). In contrast, a DEG expressed in both lines may have significant differences between the two lines (e.g., choosing a gene with a 20-fold R-line change and with a 2-fold S-line change would cause a false-negative result). We identified 5910 up-RDEGs and 3091 down-RDEGs in the R-line, which were subject to further dissection of the network involved in *Sclerotinia* resistance (Fig. 3a,b and Supplementary Table 5).

After closer inspection of these RDEGs, we found that the defense responses were more dramatic in the R-line than in the S-line. For example, the genes involved in JA and ET biosynthesis and signaling pathways, indolic GSL biosynthesis and genes encoding defense-related proteins (e.g., glucanase, chitinase, PGIP, PR2, PR3, PR4, PR5-like, PDF1.2b, lectin and osmotin) were induced more intensely in the R-line. Differences between susceptibility and resistance are likely associated with differences in the magnitude of expression changes in these defense response genes rather than with the expression of different sets of defense response genes. This difference in genes expression was further supported by the change of the chitinase, β-1,3 glucanase, PGIP activity and the change of indolic GSLs content in the R- and S-lines after *S. sclerotiorum* (Fig. 6). Interestingly, although the differences in the fold changes in RLKs, MAPK and WRKY genes between the R- and S-lines were small (Table 1 and Fig. 4), the differences in the fold changes in defense-related genes were much more dramatic (Table 2), indicating that the degree of differential expression tends to become larger between the R- and S-lines along with the defensive signal transduction chains.

ETI is the basis of the major disease resistance trait exploited for disease resistance breeding against many biotrophic and hemibiotrophic pathogens^38^. Thus far, no R-gene has been found to be associated with necrotrophic resistance, with the exception of *Arabidopsis* RLM3, a TIR domain-encoding gene involved in broad-range immunity to several necrotrophic pathogens^42^. This finding implies that ETI is not effective in necrotrophic resistance in most situations^38^. In this study, we identified 6 NBS-LRR genes that were induced in the R-line and the S-line (Supplementary Table 8). Three of these genes were up-RDEGs in the R-line. Whether these NBS-LRR genes are involved in the immune response to *S. sclerotiorum* in *B. napus* requires further studies.

PTI is a form of quantitative resistance to pathogens regardless of lifestyle^38^. *S. sclerotiorum* is an archetypical broad host-range necrotroph (BHN). In general, BHNs produce diverse PAMPs (e.g., chitin fragments) or damage-associated molecular patterns (DAMPs, e.g., oligogalacturonides) that activate PTI^38^. In this study, 57 RLK (well-characterized PRR) genes were induced in the R-line and the S-line (Supplementary Table 8). Among these genes, 19 were up-RDEGs in the R-line, including several LRR-RLK-, WAKL-, L-type lectin receptor kinase- and CRINKLY4-related genes (Table 1). Recently, a maize wall-associated kinase gene, *ZmWAK*, which confers quantitative resistance to head smut, was isolated by map-based cloning; this gene was induced after 12 h when plants were infected^43^. PRR stimulation is a key step in the early stages of PTI^44^,^45^. The induced RLKs (particularly the up-RDEGs) might play important roles in recognizing D/PAMPs produced by *S. sclerotiorum* and in triggering defense responses.

After PRR stimulation, distinct MAPK cascades are activated^10^. Our study found that the most strongly induced MAPKKK and MKK genes were several copies of *MAPKKK19* and *MKK9* in the R-line. Two *MAPKKK19* and 3 *MKK9* copies were up-RDEGs (Fig. 4). A recent study showed that MAPKKK19 interacted with MKK9 in a yeast two-hybrid system. The interaction was further confirmed by bimolecular fluorescence complementation analysis in *B. napus*^15^. Therefore, the MAPKKK19 to MKK9 pathway is likely an important signal transduction branch in the *B. napus* defense responses to *S. sclerotiorum*. Furthermore, several copies of *RAF48*, *ZIK8* and *MKK4* were identified as up-RDEGs and may be important for *Sclerotinia* resistance (Fig. 4). Rapid transcriptional induction of several copies of *WRKY6*, *8*, *11*, *15*, *28*, *33*, *40*, *69* and *75* were identified (Fig. 4). Previous studies demonstrated that the overexpression of *AtWRKY28* and *AtWRKY75* in *Arabidopsis* and *BnWRKY33* in *B. napus* markedly enhanced *S. sclerotiorum* resistance^13^,^14^. The discovery of additional WRKY genes induced by *S. sclerotiorum* infection may provide more target genes for further manipulation of the genes to improve resistance.

*B. napus* rapidly enhanced JA and ET biosynthesis and reduced SA, ABA and GA biosynthesis in response to *S. sclerotiorum* infection (Fig. 5). The JA/ET signaling pathways were also significantly induced, while the SA, ABA, GA, auxin and CK signaling pathways were noticeably inhibited (Fig. 5). Moreover, JA/ET biosynthesis and signaling pathways were more significantly induced in the R-line compared with the S-line at 48 hpi (Fig. 5). These results suggest that JA/ET contribute to resistance against *S. sclerotiorum*, coinciding with the general model that JA/ET are usually associated with the defense against necrotrophic pathogens^18^,^46^.

**Figure 8:**
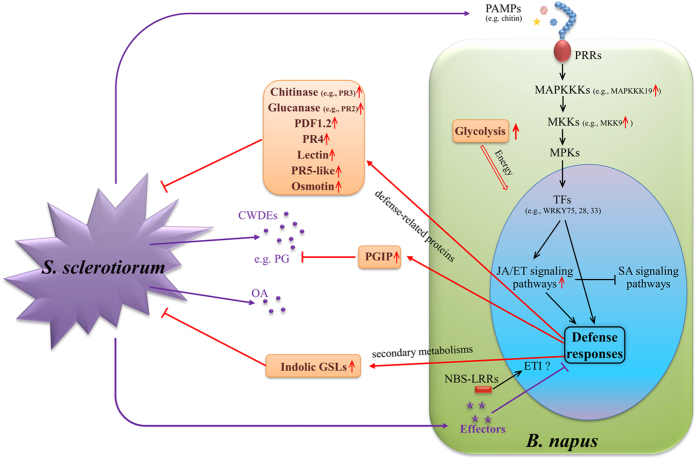
A model to describe the major molecular and physiological reactions for the defensive resistance to *S. sclerotiorum* in *B. napus*. The red upward arrows highlight the up-RDEGs involved in the respective pathways in the R-line. CWDE: cell wall-degrading enzymes; OA: oxalic acid; TF: transcription factor; PG: polygalacturonase; PGIP: polygalacturonase inhibitor protein; GSL: glucosinolate.

Based on our results, we proposed a model to describe the major molecular and physiological reactions underlying the defense responses to *S. sclerotiorum* in *B. napus* (Fig. 8). Upon the infection of *S. sclerotiorum*, P/DAMP perception and a series of signal transductions were initiated and *B. napus* activates its defense to restrict *S. sclerotiorum* development and spread by (i) synthesizing antibacterial substances to inhibit the growth of *S. sclerotiorum* (e.g., generating glucanase and chitinase to degrade the fungal cell walls (Table 2) and enhancing indolic GSL biosynthesis (Fig. 7)), and (ii) generating additional defensive enzymes to hinder *S. sclerotiorum* virulence factors (e.g., generating PGIP to inhibit PG (Table 2)).

## Methods

### Plant material and *S. sclerotiorum* resistance characterization

Both R- and S-lines were winter-type lines with similar growth periods and were grown in disease nursery plots at the experimental farm of Huazhong Agricultural University, Wuhan, China. The *S. sclerotiorum* isolate SS-1 was maintained and cultured on potato dextrose agar^7^. The phenotypic characterization of the stem resistance of these two lines to *S. sclerotiorum* was performed in three consecutive years from 2012 to 2014 using the procedure described in our previous study^7^.

### Sampling for RNA-seq

Pathogen inoculation and tissue harvest for RNA-seq were performed in 2013 following the procedures described by Zhao *et al.*^22^ with minor modifications. Plants were selected for inoculation and sampling using a randomized design with three biological replicates for both lines. Each replicate consisted of 30 plants for three time points (24, 48 and 96 hpi) and two treatments (inoculated and mock-inoculated controls). When the plants were at the termination of flowering, three sites on the primary stem were inoculated at three consecutive internodes (approximately 30–60 cm above the ground) with 7-mm diameter mycelial agar plugs punched from the growing margin of a 2-day-old culture of *S. sclerotiorum*. Mock-inoculated plants were treated with 7-mm diameter agar plugs only. Each plug was affixed with plastic wrap to ensure the close contact of the inocula with the stem surface and to maintain humidity. Each line yielded 90 inoculation sites per biological replicate or 270 sites total. Epidermal stem tissues extending 10 mm beyond the inoculation site and 1 mm deep were excised with a razor blade. Tissues harvested from one biological replicate at each time point (five individual plants comprising 15 inoculation sites) were pooled as one sample. Harvested tissues were frozen immediately in liquid nitrogen and stored at −80 °C.

### RNA extraction, cDNA library construction and RNA sequencing

Total RNA was extracted using a Plant Total RNA Extraction Kit (BioTeke, China) following the manufacturer’s instructions and treated with RNase-free DNase I (Thermo Scientific, USA) to remove genomic DNA contamination. Before RNA extraction, mock-inoculated samples from one biological replicate at each time point were pooled as a mixed mock-inoculated RNA sample. In total, 24 RNA samples (three inoculated samples at 24, 48 and 96 hpi and a mixed mock-inoculated sample for each biological replicate of each line) were subjected to library construction using an Illumina^®^ TruSeq^™^ RNA Sample Preparation Kit following the manufacturer’s instructions. All samples were sequenced using an Illumina HiSeq 2000 sequencer at the National Key Laboratory of Crop Genetic Improvement, Huazhong Agricultural University. The sequencing was performed as paired-end reads that were 2×101 bp in length. The original data set was deposited in the NCBI Sequence Read Archive (accession no. SRP053361).

### Data preprocessing, read mapping and differential gene expression analysis

Various quality controls for raw reads were conducted using the NGS QC tool kit^47^ (i) to remove the reads containing primer/adaptor sequences and the low-quality reads (the number of bases whose PHRED-like score (Q-score) was less than 20 exceeded 30%), (ii) to trim the first ten base pairs of the reads that showed unstable base composition as determined by the percentages of four different nucleotides (A, T, C, and G) and the low-quality bases (Q-score < 20) from the 3’ end of the reads, and (iii) to remove the reads less than 50 bp in length.

All high-quality reads of each sample that passed the quality control assay were mapped to the *B. napus*^32^ and *S. sclerotiorum*^35^ genomes separately by TopHat v2.0.11 using the default parameters^48^. Only uniquely mapped reads were considered for gene expression analysis. Differential gene expression and transcript abundance were calculated with the program Cufflinks v2.2.0^49^. The transcript abundance of each gene was estimated by FPKM. DEGs between inoculated and mock-inoculated samples were identified according to the restrictive conditions of an absolute value of log_2_ fold changes ≥ 1 and a FDR ≤ 0.01.

### Gene ontology and enrichment analysis

For gene function annotation, all *B. napus* genes (101,040) were searched against the NCBI non-redundant (Nr) protein database using BlastP with an E-value ≤ 1E-05. GO terms associated with each BLAST hit were annotated using Blast2GO^37^. Then, all *B. napus* genes were searched against the InterPro database (http://www.ebi.ac.uk/interpro/) using InterProScan5^50^. Finally, the GO terms of the *B. napus* genes were annotated by merging the Blast2GO and InterPro annotation results. GO enrichment analysis provided all of the GO terms that were significantly enriched in DEGs compared with the genome background using Blast2GO with a false discovery rate (FDR) ≤ 0.01. The redundancies of significantly enriched GO terms were reduced using REVIGO (similarity cutoff = 0.75)^51^. Furthermore, homologs of *Arabidopsis* genes in the *B. napus* genome were identified using the BlastP program with an E-value ≤ 1E-05, identity ≥ 50% and coverage ≥ 50%.

### Validation of RNA-seq analysis by qPCR

qPCR assays were performed to confirm the RNA-seq results. Two micrograms of total RNA from each sample (the same samples as for RNA-seq) was used to synthesize cDNA using a TransScript One-Step gDNA Remover and cDNA Synthesis Kit according to the manufacturer’s instructions (TransGen, China). The qPCR was performed as described previously^52^. The data were collected from three biological and four technical replicates. The transcript level was normalized using four reference genes (*BnaC08g12720D* (*UBC9*), *BnaA10g06670D* (*UBC10*), *BnaC09g47620D* (*YLS8*) and *BnaA09g14410D* (*PP2A-1*)), which varied little in our RNA-seq analysis and were used as reference genes in *Arabidopsis*^53^. The primers used in these experiments are listed in Supplementary Table 4.

### Measurement of enzyme activities

The stem samples were collected at 48 and 96 h from both *S. sclerotiorum*- and mock-inoculated plants with five biological replicates. The inoculation and tissue harvest methods were the same as RNA-seq. All the samples were immediately frozen and ground to fine powders in liquid nitrogen. Approximately 0.5 g powder of each sample was added to 8 volumes (~ 4 ml) of cold phosphate buffered saline (PBS, 137 mmol/L NaCl, 2.7 mmol/L KCl, 10 mmol/L Na_2_HPO_4_, 1.8 mmol/L KH_2_PO_4_, PH 7.4) and subsequently shaken at 4 °C overnight. After centrifugation (3,000 rpm) at 4 °C for 20 min, the supernatant was used as crude enzyme. Ten microliter of the supernatant was used for enzyme activity measurement by enzyme-linked immunosorbent assay (ELISA). The activity of chitinase, β-1,3 glucanase and PGIP was measured using Plant chitinase ELISA Kit, Plant β-1,3 glucanase ELISA Kit and Plant PGIPs ELISA Kit (SinoBestBio, China) following the manufacturer’s instructions, respectively.

### Glucosinolate analysis

Glucosinolates were extracted from the stem and analyzed by high-performance liquid chromatography (HPLC, Waters 2487-600-717) as described previously^54^,^55^ with minor modifications. Approximately 0.4 g stem powder of each sample (the same samples as for enzyme activity measurement) was extracted with 4 ml of boiling 90% methanol. The final volume of GSL extract was 1 ml, and 10 μl extract was measured by HPLC. Individual glucosinolates were identified by retention times and quantified using 2-propenyl glucosinolate as an internal standard as described by Feng *et al.*^55^

## Additional Information

**How to cite this article**: Wu, J. *et al.* Comparative transcriptomic analysis uncovers the complex genetic network for resistance to *Sclerotinia sclerotiorum* in *Brassica napus*. *Sci. Rep.* **6**, 19007; doi: 10.1038/srep19007 (2016).

## Supplementary Material

Supplementary Information

Supplementary Table S2

Supplementary Table S3

Supplementary Table S4

Supplementary Table S5

Supplementary Table S6

Supplementary Table S7

Supplementary Table S8

Supplementary Table S9

Supplementary Table S10

## Figures and Tables

**Table 1:** A list of RLK and NBS-LRR genes that were up-regulated RDEGs in the R-line after *S. sclerotiorum* infection.

Gene name	24 h^a^		48 h		96 h		A. thaliana Locus	Description
	S	R	S	R	S	R		
BnaA04g06520D	–	–	3.86	4.35	2.81	7.78	AT4G13920.1	Receptor like protein 50
BnaC06g28540D	–	–	–	2.97	–	–	AT1G67510.1	Leucine-rich repeat protein kinase family protein
BnaC06g32810D	–	–	–	3.86	–	2.91	AT1G71830.1	somatic embryogenesis receptor-like kinase 1
BnaCnng03730D	2.06	–	2.11	7.26	2.08	3.23	AT5G02070.1	WALL-associated receptor kinase-like 20
BnaA10g27400D	3.66	–	4.11	10.63	4.14	4.29	AT5G02070.1	WALL-associated receptor kinase-like 20
BnaA09g35490D	2.87	–	2.3	10.34	–	–	AT3G55950.1	CRINKLY4 related 3
BnaC08g26870D	2.77	–	2.64	8.06	–	–	AT3G55950.1	CRINKLY4 related 3
BnaC05g19430D	–	–	–	2.93	–	–	AT1G27190.1	Leucine-rich repeat protein kinase family protein
BnaA09g29620D	–	–	–	3.05	–	–	AT1G27190.1	Leucine-rich repeat protein kinase family protein
BnaA09g02800D	3.23	–	0.49	3.29	0.22	0.49	AT5G47850.1	CRINKLY4 related 4
BnaC04g27370D	–	–	–	3.61	–	4.99	AT3G53380.1	L-type lectin receptor kinase VIII.1
BnaA09g56900D	2.45	–	2.13	10.56	2.08	2.13	AT1G15530.1	L-type lectin receptor kinase S.1
BnaC08g38820D	2.03	–	–	7.41	–	–	AT1G15530.1	L-type lectin receptor kinase S.1
BnaA07g26080D	–	–	–	–	–	3.1	AT1G66830.1	Leucine-rich repeat protein kinase family protein
BnaA07g26070D	–	–	–	–	–	3.56	AT1G66830.1	Leucine-rich repeat protein kinase family protein
BnaC08g42220D	4.63	–	6.32	37.79	8.75	10.34	AT1G11050.1	Protein kinase superfamily protein
BnaA09g47910D	4.23	–	5.03	20.68	6.54	5.7	AT1G11050.1	Protein kinase superfamily protein
BnaA09g54750D	2.51	–	3.92	8.11	2.97	2.79	AT3G53810.1	L-type lectin receptor kinase IV.2
BnaC08g13910D	2.48	–	3.43	6.92	3.25	3.56	AT1G09970.1	Leucine-rich receptor-like protein kinase family protein
BnaC06g24010D	–	–	–	2.19	–	–	AT1G72840.2	Disease resistance protein (TIR-NBS-LRR class)
BnaA09g21180D	–	–	–	3.66	–	–	AT4G12020.3	Disease resistance protein
BnaA03g38380D	3.68	–	5.03	10.93	3.68	10.63	AT5G41750.2	Disease resistance protein (TIR-NBS-LRR class) family

^a^Fold changes relative to mock-inoculated control.

**Table 2:** Induced defense-related genes identified in the R- and S-lines after *S. sclerotiorum* inoculation.

Gene name^a^	24 h^b^		48 h		96 h		A. thaliana Locus	Description
	S	R	S	R	S	R		
BnaA01g28810D	7.2	3.8	276.3	2977.7	1509.7	1530.7	AT3G16530.1	Legume lectin
BnaC01g36130D	7.2	–	418.8	922.9	2352.5	962.1	AT3G16530.1	Legume lectin
BnaCnng78710D	12.3	–	831.7	5595.3	1595.7	1722.2	AT3G16530.1	Legume lectin
BnaA05g24230D	7.3	–	240.5	1795.3	1045.5	1618.0	AT3G16530.1	Legume lectin
BnaC04g09720D	67.2	3.0	541.2	1389.2	335.5	222.9	AT2G43590.1	Chitinase family protein
BnaA05g08640D	9.7	–	39.7	91.1	51.6	144.0	AT2G43590.1	Chitinase family protein
BnaA04g25220D	2.3	0.5	4.8	8.4	3.5	4.3	AT2G43590.1	Chitinase family protein
BnaC03g19370D	4.9	–	3.2	6.3	–	2.5	AT2G43590.1	Chitinase family protein
BnaA03g56430D	4.7	–	3.6	6.5	2.3	3.0	AT2G43590.1	Chitinase family protein
BnaCnng39650D	–	–	–	2.1	–	4.7	AT2G43590.1	Chitinase family protein
BnaC03g24290D	–	0.4	–	3.6	–	5.3	AT2G43590.1	Chitinase family protein
BnaA03g20310D	–	–	–	–	–	4.9	AT2G43590.1	Chitinase family protein
BnaA05g26640D	2.3	–	54.2	344.9	166.6	455.1	AT3G12500.1	PR3 (basic chitinase)
BnaC05g40680D	–	0.3	16.6	93.1	60.5	139.1	AT3G12500.1	PR3 (basic chitinase)
BnaC03g33880D	7.1	–	132.5	86.2	278.2	82.7	AT3G04720.1	PR4 (Hevein-like protein)
BnaC03g33890D	2.2	0.4	10.9	17.3	13.0	17.6	AT3G04720.1	PR4 (Hevein-like protein)
BnaC03g33900D	–	–	17.1	86.8	88.0	245.6	AT3G04720.1	PR4 (Hevein-like protein)
BnaA03g28780D	2.1	0.3	28.1	177.3	88.6	382.7	AT3G04720.1	PR4 (Hevein-like protein)
BnaA03g28770D	2.2	–	6.6	16.8	7.8	20.0	AT3G04720.1	PR4 (Hevein-like protein)
BnaA03g28760D	4.1	–	87.4	–	96.3	–	AT3G04720.1	PR4 (Hevein-like protein)
BnaA09g44230D	15.8	–	29.2	342.5	21.6	76.6	AT1G19320.1	PR5-like
BnaC05g14950D	–	0.2	4.1	23.6	10.5	37.0	AT1G19320.1	PR5-like
BnaA07g32130D	–	–	5.6	6.2	16.0	11.3	AT2G26020.1	PDF1.2b
BnaA01g17540D	–	–	64.9	831.7	498.0	2288.2	AT4G16260.1	β-1,3-endoglucanase
BnaC01g21880D	–	–	39.9	190.0	238.9	843.4	AT4G16260.1	β-1,3-endoglucanase
BnaC08g28170D	–	0.4	–	5.0	–	4.9	AT3G57260.1	PR2 (β-1,3-glucanase 2)
BnaC08g28150D	–	0.3	–	5.7	–	4.7	AT3G57260.1	PR2 (β-1,3-glucanase 2)
BnaC09g48700D	3.0	–	50.9	153.3	107.6	151.2	AT5G06860.1	PGIP
BnaA10g24090D	–	–	24.8	73.0	39.7	52.3	AT5G06860.1	PGIP
BnaC09g48690D	–	–	–	10.8	17.9	19.0	AT5G06860.1	PGIP
BnaC09g48680D	–	–	–	25.5	25.8	20.3	AT5G06860.1	PGIP
BnaA10g24050D	0.4	–	4.5	8.8	41.1	75.1	AT5G06860.1	PGIP
BnaA10g24060D	–	–	–	2.1	5.4	5.1	AT5G06860.1	PGIP
BnaA10g24080D	–	–	–	4.7	11.0	13.9	AT5G06860.1	PGIP
BnaA02g21660D	–	–	5.6	174.9	22.8	3281.2	AT4G11650.1	osmotin 34

^a^Genes labeled with underscore were up-RDEGs.

^b^Fold changes relative to mock-inoculated control.
