# Corrigendum: Comparative transcriptomic analysis uncovers the complex genetic network for resistance to *Sclerotinia sclerotiorum* in *Brassica napus*

**DOI:** 10.1038/srep42829

**Published:** 2017-02-21

**Authors:** Jian Wu, Qing Zhao, Qingyong Yang, Han Liu, Qingyuan Li, Xinqi Yi, Yan Cheng, Liang Guo, Chuchuan Fan, Yongming Zhou

Scientific Reports 6: Article number: 19007; 10.1038/srep19007 published online: 01 08 2016; updated: 02 21 2017.

The original version of this Article contained an incorrect version of Supplementary Table S7. This error has now been corrected in the HTML version of this Article; the PDF version was correct at the time of publication.

