# Supplementary material for: Comparative transcriptomic analysis uncovers the complex genetic network for resistance to Sclerotinia sclerotiorum in Brassica napus
Source: Sci Rep. 2016 Jan 8;6:19007. doi: 10.1038/srep19007 (PMC4705546; doi:10.1038/srep19007)
Supplement: Supplementary Information [file srep19007-s1.pdf]

Comparative transcriptomic analysis uncovers the complex genetic network for resistance to *Sclerotinia sclerotiorum* in *Brassica napus*

Jian Wu, Qing Zhao, Qingyong Yang, Han Liu, Qingyuan Li, Xinqi Yi, Yan Cheng, Liang Guo, Chuchuan Fan, Yongming Zhou*

National Key Laboratory of Crop Genetic Improvement, Huazhong Agricultural University, Wuhan 430070, China

**Correspondence: Y. Zhou. e-mail: ymzhou@mail.hzau.edu.cn*

Additional Information

Competing financial interests:

The authors have no competing interests as defined by Nature Publishing Group, or other interests that might be perceived to influence the results and/or discussion reported in this paper.

Supplementary information:

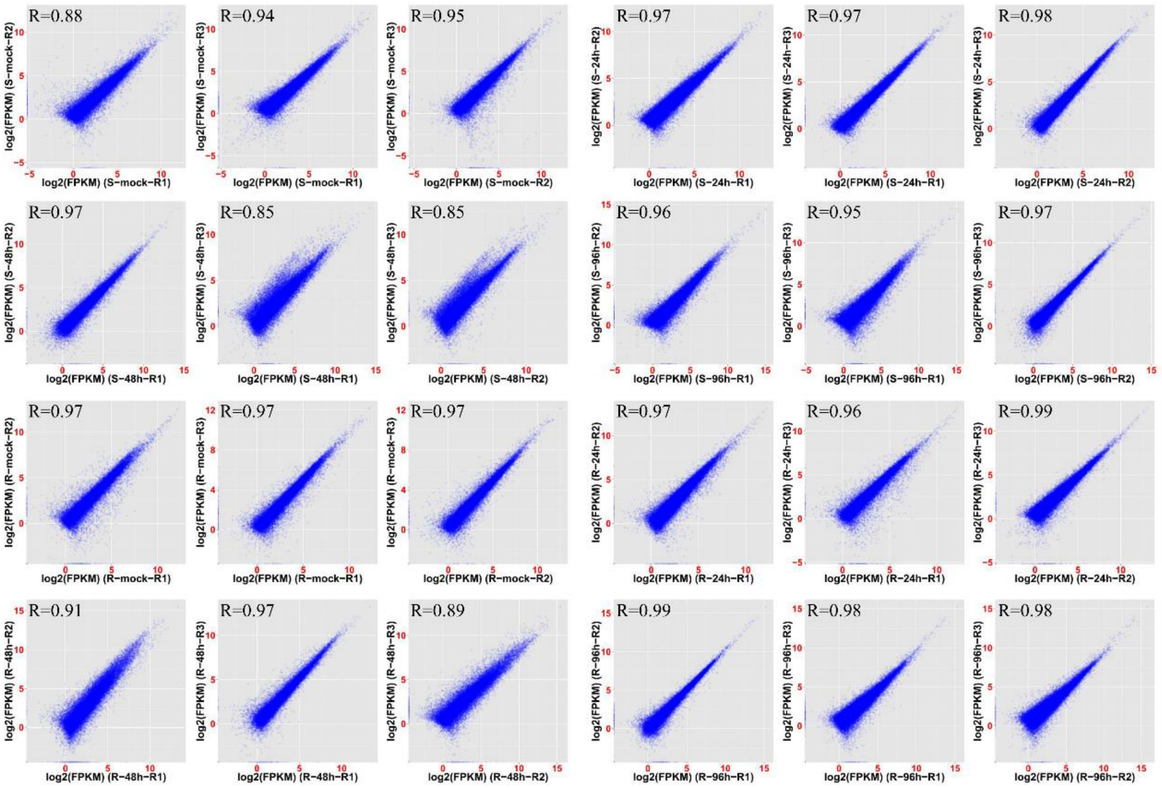

Supplementary Figure 1. Pearson correlation coefficients between each pair of biological replicates in different sampling conditions for both R- and S-lines.

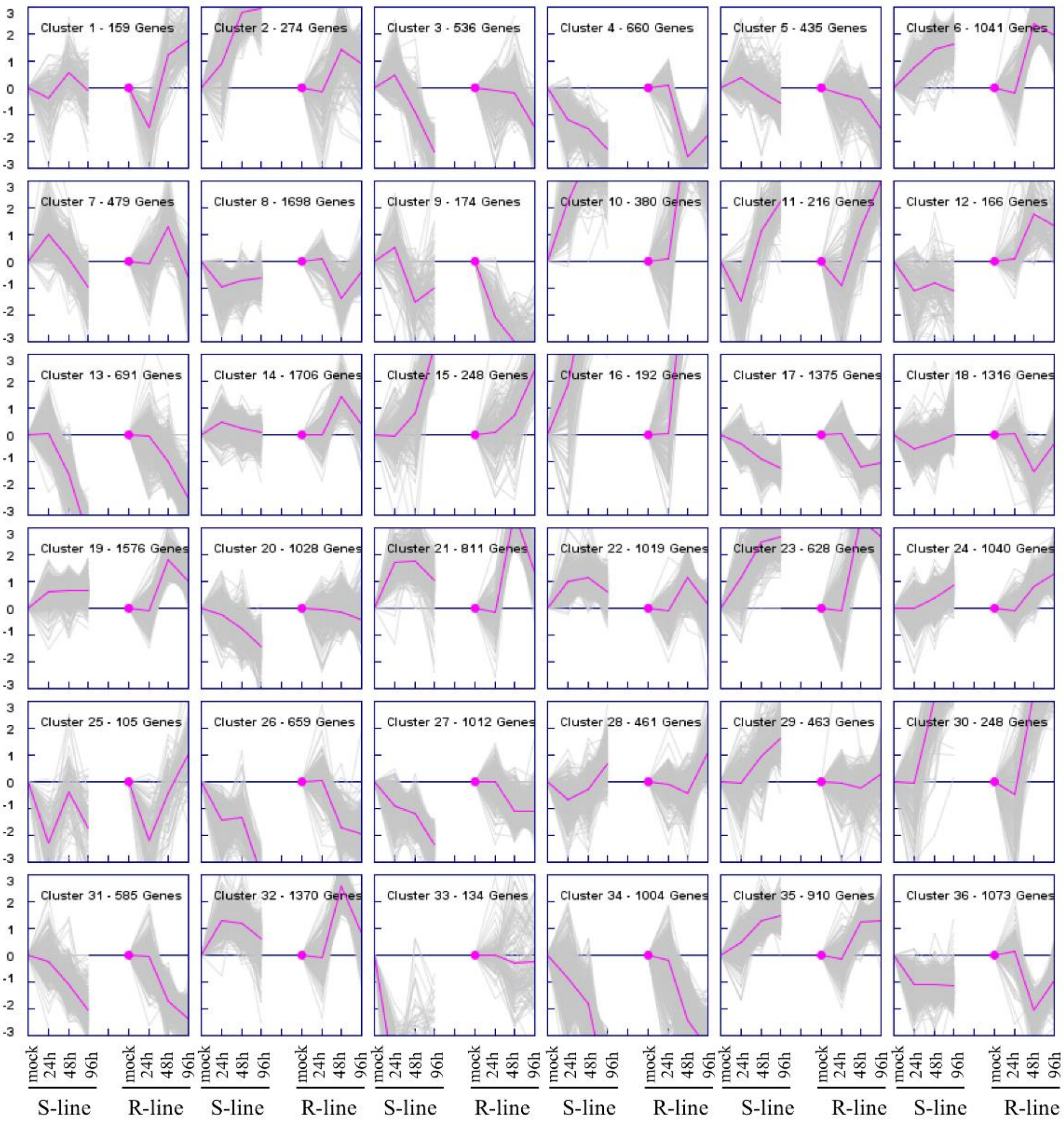

Supplementary Figure 2. Cluster analysis of all 25,872 R- and S-lines DEGs after *S. sclerotiorum* infection. The 36 clusters were identified by Genesis based on the K-means clustering method. The y-axis represents the $\log_2$ fold change (inoculated/mock-inoculated).

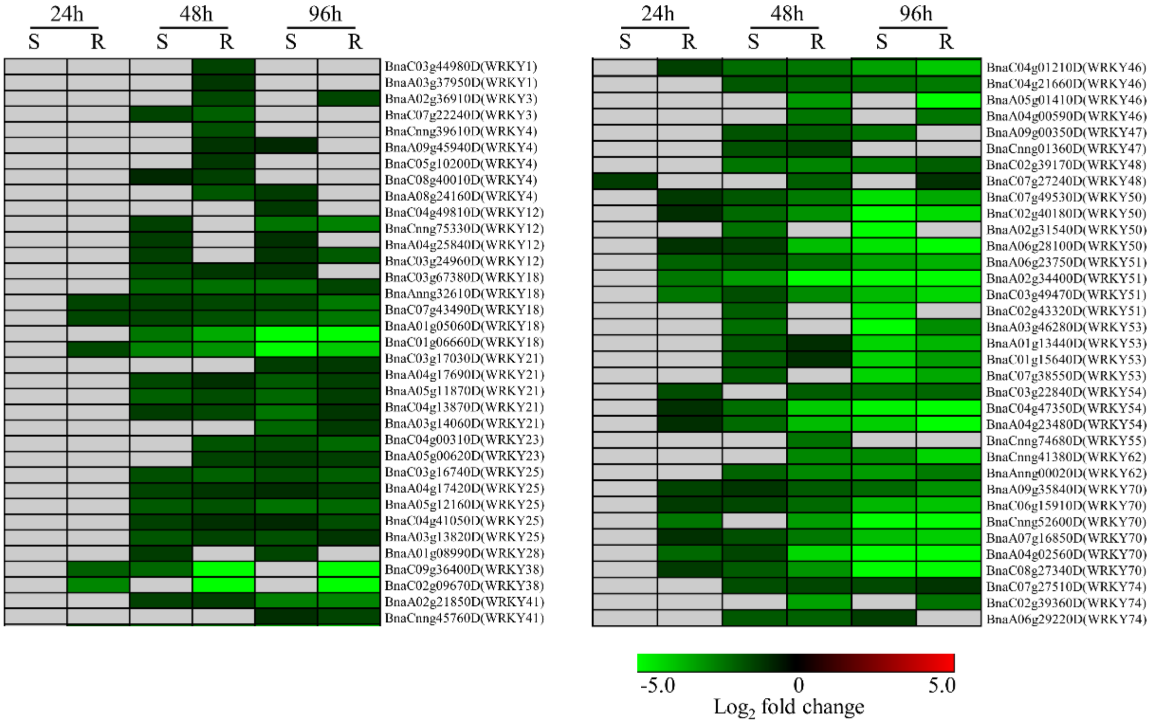

Supplementary Figure 3. WRKY transcription factors down-regulated in the R- and S-lines after inoculation with *S. sclerotiorum*.

Supplementary Table 1. Statistics of RNA-seq reads and mapped reads.

Samples [a]	Total raw reads	Total clean reads	Total clean nucleotides (bp)	Q30	Mapped reads to Bn genome sequence [b]	Uniquely mapped reads to Bn genome sequence	Mapped reads to Ss genome sequence [c]
S-mock-R1	32,100,032	31,490,234	2,832,443,214	98.80%	82.20%	73.10%	0.00%
S-mock-R2	42,806,216	41,628,406	3,752,300,064	98.10%	87.40%	78.90%	0.00%
S-mock-R3	49,993,096	49,048,014	4,456,343,912	99.40%	90.00%	82.30%	0.00%
S-24h-R1	36,203,074	35,163,910	3,161,378,333	98.00%	85.80%	77.55%	1.40%
S-24h-R2	57,555,374	56,100,348	5,044,159,554	98.20%	86.20%	77.70%	1.50%
S-24h-R3	37,455,690	36,532,782	3,285,054,681	98.20%	86.40%	77.95%	0.80%
S-48h-R1	43,784,384	42,609,698	3,841,428,592	97.00%	79.60%	70.30%	3.70%
S-48h-R2	40,017,584	38,976,116	3,503,214,527	98.00%	85.60%	76.50%	2.20%
S-48h-R3	37,682,792	36,895,696	3,322,793,527	98.80%	84.70%	75.50%	2.10%
S-96h-R1	41,386,668	40,516,120	3,646,456,449	98.80%	66.50%	56.70%	16.30%
S-96h-R2	48,377,772	47,335,534	4,255,438,571	98.70%	71.80%	62.25%	16.50%
S-96h-R3	24,754,582	24,246,052	2,185,804,131	98.90%	75.80%	65.80%	10.90%
R-mock-R1	23,558,100	22,616,466	2,050,784,963	97.60%	90.10%	82.00%	0.00%
R-mock-R2	25,596,942	24,468,302	2,218,342,431	97.30%	89.50%	81.30%	0.00%
R-mock-R3	60,262,066	57,530,532	5,215,751,636	97.20%	89.90%	82.00%	0.00%
R-24h-R1	40,397,800	38,559,788	3,495,457,564	97.10%	90.20%	82.30%	0.30%
R-24h-R2	31,640,890	30,188,824	2,736,243,587	97.20%	90.10%	82.50%	0.60%
R-24h-R3	30,641,966	29,308,510	2,657,171,980	97.30%	88.90%	80.80%	1.30%
R-48h-R1	59,434,680	56,912,906	5,159,671,481	97.20%	88.70%	80.35%	1.40%
R-48h-R2	40,199,342	37,997,064	3,442,310,506	96.80%	87.90%	80.65%	2.90%
R-48h-R3	24,378,982	23,219,602	2,104,179,927	97.10%	89.50%	81.95%	1.30%
R-96h-R1	22,375,572	21,391,248	1,938,778,497	97.20%	87.60%	78.80%	3.10%
R-96h-R2	34,026,644	32,453,868	2,941,367,741	97.10%	86.80%	78.20%	4.20%
R-96h-R3	31,699,636	30,292,696	2,745,947,360	97.20%	85.80%	76.10%	4.70%
Total	916,329,884	885,482,716	79,992,823,228	-	-	-	-
Average	38,180,412	36,895,113	3,333,034,301	97.80%	85.30%	76.70%	3.10%

[a] S S-line; R R-line; R1, R2 and R3 three biological replicates

[b] Bn *Brassica napus*

c Ss *Sclerotinia sclerotiorum*

Supplementary Table 2. Gene transcript abundances of mock samples of R- and S-lines were estimated by FPKM using Cufflinks. An FPKM cutoff value of 1 was set to declare a gene expressed.

Supplementary Table 3. DEGs identified from R- and S-lines at 24, 48 and 96 h after inoculation with *S. sclerotiorum*.

Supplementary Table 4. Primer sequences used for qPCR analysis.

Supplementary Table 5. RDEGs identified from the R-line compared with the S-line at 24, 48 and 96 h after inoculation with *S. sclerotiorum*.

Supplementary Table 6. GO enrichment analysis of up-RDEGs and down-RDEGs using Blast2GO. Three major functional categories were grouped: biological process (P), cellular component (C) and molecular function (F).

Supplementary Table 7. RLKs, MAPKKK, MKK, MPK and WRKY genes identified in the *B. napus* genome according to the homologous genes in *Arabidopsis*.

Supplementary Table 8. RLK genes and NBS-LRR genes induced in R- and S-lines after inoculation with *S. sclerotiorum*.

Supplementary Table 9. Genes involved in the SA, JA, ET, ABA, GA, auxin and CK biosynthesis and signaling pathways identified in *B. napus* according to the homologous genes in *Arabidopsis*.

Supplementary Table 10. Genes for GSL biosynthesis identified in *B. napus* according to the homologous genes in *Arabidopsis*.

Supplementary Table 11. Analytical data of two aliphatic and three indolic glucosinolates detected before and after *S. sclerotiorum* infection.

Samples	Indolic GSLs (nmol/g)				Aliphatic GSLs			Total GSLs
	4-hydroxy-indol-3-ylmethyl	Indol-3-ylmethyl	4-methoxy-indol-3-ylmethyl	Total	2-hydroxy-3-Butenyl	2-hydroxy-4-Pentenyl	Total	
S-48h-mock	17.21 ± 3.20	15.14 ± 3.46	13.43 ± 0.96	45.78 ± 3.50	8.47 ± 0.73	22.54 ± 5.21	31.01 ± 5.09	76.79 ± 7.86
S-48h-I	11.20 ± 0.45	22.00 ± 3.96	12.4 ± 0.99	45.62 ± 4.66	n.d.	15.25 ± 8.67	15.25 ± 8.67	60.87 ± 13.30
R-48h-mock	7.66 ± 1.67	4.95 ± 1.00	5.91 ± 0.96	18.52 ± 2.71	n.d.	n.d.	-	18.52 ± 2.71
R-48h-I	7.12 ± 2.18	11.76 ± 4.36	11.67 ± 0.87	30.55 ± 5.82	n.d.	n.d.	-	30.55 ± 5.82
S-96h-mock	20.09 ± 5.24	15.50 ± 3.63	10.51 ± 2.96	46.10 ± 10.33	4.15 ± 0.92	27.08 ± 8.2	31.23 ± 8.97	77.33 ± 16.32
S-96h-I	4.87 ± 0.55	59.13 ± 6.73	29.51 ± 4.6	93.51 ± 7.81	0.69 ± 1.38	12.13 ± 4.42	12.82 ± 5.63	106.33 ± 12.60
R-96h-mock	10.01 ± 2.39	6.06 ± 1.00	3.25 ± 0.55	19.31 ± 3.29	n.d.	n.d.	-	19.31 ± 3.29
R-96h-I	10.15 ± 2.77	78.70 ± 13.62	66.09 ± 16.18	154.93 ±3 0.44	n.d.	n.d.	-	154.93 ± 30.44

Data are presented as Mean ± SD (n=5). n.d.: not detected
